# Associations of CT-Derived Body Composition with Textbook Outcomes and Early Death After Hepatectomy for Hepatocellular Carcinoma

**DOI:** 10.3390/jcm15197547

**Published:** 2026-09-28

**Authors:** Chuwen Chen, Qiwen Xiang, Jing Hu, Guoxin Chen, Hankui Hu

**Affiliations:** 1 Division of Vascular Surgery, Department of General Surgery, West China Hospital, Sichuan University, Chengdu 610106, China; 2 Department of Operating Room, West China Hospital, Sichuan University, Chengdu 610106, China; 3 Department of Health Management Centre, West China Fourth Hospital, Sichuan University, Chengdu 610106, China; 4 Department of Anesthesiology, West China Hospital, Sichuan University, Chengdu 610106, China

**Keywords:** body composition, textbook outcome, early death, hepatocellular carcinoma, hepatectomy

## Abstract

**Background:** The aim of this study was to explore associations between CT-derived body composition parameters and textbook outcomes (TOs) and early death (within one year) after hepatectomy for hepatocellular carcinoma (HCC). **Methods:** Multiple CT-derived body composition parameters were evaluated in an exploratory analysis. Sex-specific, cohort-derived cutoff values were determined using receiver operating characteristic curve analyses. Logistic and Cox regression models were used to examine associations with failure to achieve TOs and survival outcomes, respectively. No individual body composition parameter was prespecified as the primary exposure or prioritized a priori for comparative analysis. **Results:** A total of 815 patients were included in our study. The multivariate logistic regression model revealed that a low psoas muscle index (PMI, cutoff values: 7.81 cm^2^/m^2^ in males and 3.84 cm^2^/m^2^ in females) and a high subcutaneous fat index (SFI, cutoff values: 56.75 cm^2^/m^2^ in males and 80.09 cm^2^/m^2^ in females) were independently associated with failure to achieve TOs. Patients were further divided into three groups: no risk factor (*N* = 231), one risk factor (*N* = 516), and two risk factors (*N* = 68). As the number of body composition risk factors increased, the incidence of non-TOs showed a significant increasing trend (*p* for trend <0.001), and the number of body composition risk factors remained independently associated with non-TOs (1 risk factor, OR: 1.99, 95% CI: 1.33–2.97, *p* < 0.001; 2 risk factors, OR: 3.54, 95%CI: 1.91–6.58, *p* < 0.001). The 1-, 3-, and 5-year rates were 95.0%, 83.1%, and 74.1% in patients with no risk factor, 88.5%, 77.1%, and 67.5% in patients with one risk factor, and 85.3%, 70.6%, and 61.8% in patients with two risk factors, respectively. Patients with different numbers of body composition risk factors showed significant differences in survival for early death (*p* = 0.009), whereas no statistically significant difference was observed for late death (*p* = 0.627). In the multivariate Cox analysis, the number of body composition risk factors remained independently associated with early death (1 risk factor, HR: 2.02, 95% CI: 1.05–3.87, *p* = 0.035; 2 risk factors, HR: 3.32, 95% CI: 1.36–8.06, *p* = 0.008). **Conclusions:** Our findings demonstrate that low PMI and high SFI were associated with failure to achieve TOs after hepatectomy for HCC. An increasing number of these two body composition factors showed graded associations with both non-TOs and early mortality, but not with late mortality. These findings warrant validation in independent cohorts.

## 1. Introduction

Hepatectomy is an important treatment strategy for hepatocellular carcinoma (HCC) patients to achieve long-term survival [1]. However, resection of the liver is a surgical procedure with extremely high risk, which means that establishing a rigorous surgical quality assessment system is necessary to ensure optimal patient outcomes [2]. Some traditional surgical outcome parameters, such as 30-day readmission, 90-day mortality, major complications, and prolonged length of hospital stay, have been widely used to assess the quality of liver surgery [3,4]. Although a single parameter can assess the quality of liver surgery to some extent, owing to the complexity of liver surgery, a single parameter alone cannot fully reflect the multidimensional characteristics of the whole process of liver surgery. Therefore, textbook outcomes (TOs), as a composite parameter, have been proposed for a more comprehensive assessment of surgical quality [5,6]. Moreover, some recent studies have demonstrated that TOs are associated with superior prognosis in HCC patients after hepatectomy [7,8,9]. Thus, identifying risk factors for TOs may help clinicians assess surgical risk and surgical quality, make individualized treatment decisions, and maximize patient benefit.

Body composition includes adipose and muscle tissue, which to some extent reflects the nutritional and metabolic state of the organism [10]. Some relevant evaluation parameters include skeletal muscle tissue, subcutaneous adipose tissue, and visceral adipose tissue. Some recent studies investigated the relationship between body composition and the prognosis of HCC patients, reporting some interesting results. Labeur et al. reported that low skeletal muscle and low total adipose tissue were independently associated with the overall survival of HCC patients treated with sorafenib [11]. Both visceral adipose tissue and subcutaneous adipose tissue may influence the survival outcomes of HCC patients after hepatectomy [12,13]. However, current relevant studies mainly focus on the relationship between body composition and the long-term prognosis of HCC patients, and few studies have explored its relationship with short-term outcomes. In terms of quality assessment of liver surgery for HCC patients, TOs, comprising multiple surgical outcome parameters, have attracted increasing attention in recent years, but relevant studies have focused mainly on the effects of intraoperative conditions, tumor load, liver function, and other factors on TOs, while somewhat neglecting the role of individualized body characteristic parameters [14,15]. Furthermore, in addition to long-term survival, the short-term survival (e.g., early death within one year) of HCC patients after hepatectomy is also a major concern for surgeons. Therefore, the aim of this study was to explore the associations of a range of CT-derived body composition parameters with textbook outcomes and early mortality after hepatectomy for HCC and to identify candidate body composition factors for further validation. No individual body composition parameter was designated a priori as the primary exposure.

## 2. Materials and Methods

### 2.1. Patients

This retrospective study included 815 HCC patients with BCLC stage 0/A/B who underwent hepatectomy between May 2014 and May 2019 at West China Hospital (WCH) for final analysis. The exclusion criteria were as follows: (1) insufficient baseline clinical data; (2) concomitant surgery, e.g., splenectomy; (3) spontaneous HCC rupture; (4) patients with other malignant diseases; (5) Child–Pugh grade B; and (6) BCLC stage C (Figure 1). All baseline patient information was retrospectively acquired from the WCH’s Hospital Information System (HIS). This study received ethical approval from the Institutional Review Board of West China Hospital, Sichuan University.

### 2.2. Outcomes and Definitions

The primary outcome of our study were textbook outcomes (TOs), defined as R0 resection, no 30-day major morbidity (≥Clavien–Dindo grade 3), no 30-day readmission, no 90-day mortality, no perioperative blood transfusion, no prolonged hospital stay (LOS > 75th percentile, 11 days for laparoscopic hepatectomy and 13 days for open hepatectomy), and no perioperative conversion from laparoscopic to open hepatectomy [6,16]. The secondary outcome was early death, defined as death because of any cause within one year after hepatectomy [17,18]. In the early death analysis, deaths occurring within 12 months were treated as events at the date of death, whereas patients who were alive at 12 months were administratively censored at 12 months. Late death was evaluated using a 1-year landmark analysis. Only patients who were alive at 12 months after hepatectomy were included in this analysis. Follow-up for late death began at the 12-month landmark and ended at death or the last follow-up date; deaths occurring within the first postoperative year were not included in the late-mortality risk set. Overall survival (OS) was defined as the time from surgery to death from any cause or the last follow-up.

The albumin–bilirubin (ALBI) grade was used to estimate the liver function status in our study. The ALBI grades were categorized into three levels: Grade I, Grade II, and Grade III, which correspond to ALBI scores of ≤−2.60, >−2.60 to ≤−1.39, and >−1.39, respectively. The ALBI score was calculated using the formula [(log10 bilirubin (in µmol/L) × 0.66) + (albumin (in g/L) × −0.085)] [19]. The aspartate aminotransferase to platelet ratio index (APRI) and fibrosis-4 index (FIB-4) are both non-invasive markers to estimate liver cirrhosis. The APRI was calculated based on the following formula: AST (U/L)/PLT (10^9^/L) × 100. The FIB-4 was calculated based on the following formula: age × AST (U/L)/PLT (U/L) × ALT (U/L)^1/2^. Based on previous studies, the cutoff values for the APRI and FIB-4 were 1.5 and 4, respectively [20]. Prognostic nutritional index (PNI) was calculated based on the following formula: serum albumin (g/L) + 5 × lymphocyte count (10^9^/L). The cutoff value of the PNI was 45 according to previous studies [21]. Major hepatectomy was defined as the resection of more than three liver segments [22].

### 2.3. Body Composition Analysis

Body composition measurements were obtained from a single axial CT image at the third lumbar vertebral (L3) level using CoreSlicer, a web-based computer-assisted segmentation platform [23]. Previous studies have demonstrated good overall agreement and reproducibility of CoreSlicer version 1.0 (McGill University, Montreal, QC, Canada) measurements compared with established body composition analysis software [10,11]. All segmentations included in the final analytic cohort were visually reviewed by two investigators trained in L3-level body composition analysis before the measurements were finalized. The review was conducted as a consensus-based quality-control procedure rather than as independent duplicate segmentation. Any segmentation considered anatomically inaccurate was manually corrected, and differences in judgment were resolved through joint review and consensus. Manual correction was not separately recorded at the individual-scan level, and formal interobserver agreement was therefore not assessed. Patients with inadequate CT image quality or without an evaluable L3 image were excluded. Muscle tissue (−29 to 150 HU) and adipose tissue (−190 to −30 HU) were distinguished by the tissue-specific Hounsfield unit (HU), and the cross-sectional areas were calculated separately (Figure 2) [24,25]. To standardize each body composition cross-sectional area, we divided each area by the square of its height (cm^2^/m^2^). Body composition parameters after standardization included the psoas muscle index (PMI), skeletal muscle index (SMI), total SMI, subcutaneous fat index (SFI), and visceral fat index (VFI). The visceral-fat-to-muscle ratio was calculated as the visceral fat area divided by the total skeletal muscle area. The subcutaneous-fat-to-muscle ratio was calculated as the subcutaneous fat area divided by the total skeletal muscle area, and the visceral-to-subcutaneous-fat ratio was calculated as the visceral fat area divided by the subcutaneous fat area. In our study, skeletal muscle density (SMD), automatically generated by the software, was used to assess the extent of muscle fat degeneration (psoas SMD, wall SMD, and total SMD) [24].

### 2.4. Statistical Analyses

After application of the prespecified inclusion and exclusion criteria, all patients included in the final analytic cohort had complete data for the baseline variables used in the primary analysis. Therefore, no imputation was performed. The means plus standard deviations (SDs) or medians and interquartile ranges were used to describe continuous variables. The *t*-test or Mann–Whitney test was used to compare continuous variables according to the normality test. Categorical variables were expressed as frequencies and percentages, with comparisons performed using the chi-square or Fisher’s exact test. Sex-specific cutoff values for each body composition parameter were derived from receiver operating characteristic (ROC) curve analyses, using failure to achieve textbook outcomes (non-TOs) as the outcome of interest. The optimal cutoff for each parameter was selected using the maximum Youden index. No individual body composition parameter was prespecified as the primary exposure or prioritized a priori for comparative analysis. To assess the internal stability and potential optimum of the sex-specific PMI and SFI cutoffs, internal validation was performed using 2000 nonparametric patient-level bootstrap resamples, stratified by sex. Within each bootstrap sample, the sex-specific cutoff was re-estimated by maximizing the Youden index, while preserving the risk direction used in the primary analysis (lower PMI and higher SFI predicting non-TOs). The optimal score was calculated as the difference between bootstrap-sample performance and test performance in the original cohort, and was averaged across all resamples. Cutoff stability was summarized using the bootstrap median and the 2.5th–97.5th percentile interval. Optimal correction of the unadjusted odds ratios was performed on the log-odds scale and subsequently exponentiated. The Kaplan–Meier method was used to analyze survival outcomes. The multivariate Cox regression model was performed to identify variables independently associated with survival outcomes. The multivariate logistic regression model was used to identify variables independently associated with TOs. Unadjusted trend tests for binary outcomes were conducted using the univariate logistic regression model. Statistical analysis was performed using R software (version 4.2.3). Statistical significance was defined as *p* < 0.05 (two-tailed tests).

## 3. Results

### 3.1. Patient Characteristics

Our study included 815 HCC patients with BCLC stage 0/A/B who underwent hepatectomy (Figure 1). The rate of postoperative outcomes meeting the TO criteria was 71.9% across the entire cohort. Figure 3 shows the specific proportions of each type of non-TO. A total of 182 (22.3%) patients experienced a prolonged LOS, 27 (3.3%) patients received perioperative blood transfusion, 23 (2.8%) patients experienced major complications (≥Clavien–Dindo grade 3), 10 (1.2%) patients experienced 30-day readmission after discharge, 15 (1.8%) patients experienced death within 90 days after hepatectomy, 8 (1.0%) patients experienced intraoperative conversion from LLR, and 4 (0.5%) patients underwent non-R0 resection.

Supplementary Table S1 presents the differences in body composition variables stratified by sex. The median imaging-to-surgery interval was 4 days (IQR, 2–9 days; range, 0–29 days) for the entire cohort (Figure S1). Compared with female patients, male patients had a higher PMI (*p* < 0.001), a higher wall SMI (*p* < 0.001), a higher wall SMD (*p* < 0.001), a higher total SMI (*p* < 0.001), a higher total SMD (*p* < 0.001), a lower SFI (*p* < 0.001), a lower visceral-fat-to-muscle ratio (*p* < 0.001), a lower subcutaneous-fat-to-muscle ratio (*p* = 0.018), and a higher visceral-to-subcutaneous-fat ratio (*p* < 0.001). For further analysis, we utilized ROC curves to obtain the best sex-specific cutoff values for each body composition variable for predicting TOs, and converted each body composition variable into a dichotomous variable based on the cutoffs (High level, > cutoff value; Low level, ≤ cutoff value). The cutoff values were 7.81 cm^2^/m^2^ for male patients and 3.84 cm^2^/m^2^ for female patients regarding the PMI, 58.80 HU for male patients and 49.44 HU for female patients regarding psoas SMD (Figure S2A–D), 45.43 cm^2^/m^2^ for male patients and 38.45 cm^2^/m^2^ for female patients concerning wall SMI, 53.54 HU for male patients and 36.31 HU for female patients for wall SMD (Figure S3A–D), 50.97 cm^2^/m^2^ for male patients and 44.34 cm^2^/m^2^ for female patients regarding total SMI, 57.57 HU for male patients and 37.70 HU for female patients concerning total SMD (Figure S4A–D), 56.75 cm^2^/m^2^ for male patients and 80.09 cm^2^/m^2^ for female patients for the SFI, 51.46 cm^2^/m^2^ for male patients and 26.89 cm^2^/m^2^ for female patients regarding the VFI (Figure S5A–D), 0.96 for male patients and 2.31 for female patients concerning the subcutaneous-fat-to-muscle ratio, 1.06 for male patients and 0.96 for female patients for the visceral-fat-to-muscle ratio (Figure S6A–D), and 0.65 for male patients and 0.49 for female patients regarding the visceral-to-subcutaneous-fat ratio (Figure S7A,B).

Based on whether TOs were achieved, we further divided patients into a TO group (*N* = 586) or a Non-TO group (*N* = 229). Compared with patients in the TO group, patients in the Non-TO group had more advanced tumor stages (*p* = 0.011), larger tumor sizes (*p* < 0.001), higher proportions of more than 400 ng/mL of α-fetoprotein (*p* = 0.018) and ALBI grade II (*p* < 0.001), and higher levels of ALT (*p* = 0.003), AST (*p* < 0.001), APRI (*p* = 0.004), and FIB-4 (*p* = 0.045). Nonetheless, patients in the Non-TO group had lower levels of albumin (*p* < 0.001) and PNI (*p* < 0.001). A higher proportion of patients in the Non-TO group underwent major hepatectomy (*p* < 0.001), and patients in the Non-TO group had more intraoperative bleeding (*p* < 0.001) and longer hospital stays (*p* < 0.001, Table 1).

Compared with patients in the Non-TO group, a higher proportion of patients in the TO group had high levels of PMI (*p* < 0.001), psoas SMD (*p* = 0.049), and wall SMD (*p* = 0.013), and a lower proportion of patients had high levels of SFI (*p* = 0.015) and a higher subcutaneous-fat-to-muscle ratio (*p* = 0.016, Table 1).

### 3.2. The Association Between Body Composition and Failure to Achieve TOs

To further identify body composition variables independently associated with non-TOs, we performed univariate and multivariate logistic regressions. Variables potentially independently associated with non-TOs in the univariate logistic regression were further included in the multivariate logistic regression model (Table 2). In our multivariate logistic regression model, a low PMI (OR: 1.91, 95% CI: 1.33–2.73, *p* < 0.001) and a high SFI (OR: 1.91, 95% CI: 1.25–2.92, *p* = 0.003) were independent risk variables for the achievement of TOs. Other variables independently associated with non-TOs included α-fetoprotein (400 ng/mL, OR: 1.41, 95% CI: 1.01–1.97, *p* = 0.046), AST (40 U/L, OR: 1.60, 95% CI: 1.14–2.26, *p* < 0.001), albumin (40 g/L, OR: 0.53, 95% CI: 0.36–0.77, *p* = 0.001), major hepatectomy (OR: 1.60, 95% CI: 1.14–2.24, *p* = 0.006), and intraoperative blood loss (400 mL, OR: 1.66, 95% CI: 1.10–2.50, *p* = 0.016, Table 2).

To assess the feasibility of interaction analyses, we examined the distribution of patients and non-TO events across clinically relevant strata. Non-TOs were observed in 176 of 694 patients (25.4%) with ALBI grade I and in 53 of 121 patients (43.8%) with ALBI grade II. The corresponding numbers were 114 of 510 patients (22.4%) undergoing non-major hepatectomy and 115 of 305 patients (37.7%) undergoing major hepatectomy. With respect to tumor burden, non-TOs occurred in 209 of 764 patients (27.4%) with BCLC stage 0/A and in 20 of 51 patients (39.2%) with BCLC stage B. Further cross-classification by body composition exposure yielded sparse event cells, particularly among patients with BCLC stage B. Formal interaction analyses were therefore not undertaken, as they were unlikely to provide sufficiently precise or stable estimates.

### 3.3. Exploratory Associations of the Number of Unfavorable Body Composition Factors with Outcomes

Because a low PMI and a high SFI were the two body composition parameters that retained statistically significant associations in the exploratory multivariable analysis, we performed a post hoc analysis according to the number of these unfavorable factors. Based on the number of body composition risk factors, we categorized patients into three groups: no risk factor (high PMI and low SFI, *N* = 231), one risk factor (low PMI or high SFI, *N* = 516), and two risk factors (low PMI and high SFI, *N* = 68). Table 3 shows the results of the comparison of each specific type of non-TO between patients with or without body composition risk factors. Patients with body composition risk factors had significantly higher rates of 30-day major morbidity (*p* = 0.034), 30-day readmission (*p* = 0.045), 90-day mortality (*p* = 0.014), perioperative blood transfusion (*p* = 0.043), and prolonged LOS (*p* = 0.006). Patients with no, one, and two risk factors had non-TO rates of 17.3%, 30.8%, and 44.1%, respectively. As the number of risk factors increased, the incidence of non-TOs showed a significant increasing trend (Figure 4, *p* for trend <0.001). We also included the number of risk factors as a variable in the multivariate model for adjustment (Table 3). In our model, the number of body composition risk factors remained independently associated with non-TOs (1 risk factor, OR: 1.99, 95% CI: 1.33–2.97, *p* < 0.001; 2 risk factors, OR: 3.54, 95% CI: 1.91–6.58, *p* < 0.001, Table S2).

### 3.4. Impact of the Number of Body Composition Risk Factors on Early Death (Within One Year After Hepatectomy)

In the entire cohort, the median follow-up time was 57 months. During the follow-up period, 245 deaths occurred, including 79 early deaths within 1 year after hepatectomy and 166 late deaths among patients who survived beyond 1 year. The 1-, 3-, and 5-year rates were 95.0%, 83.1%, and 74.1% in patients with no risk factor, 88.5%, 77.1%, and 67.5% in patients with one risk factor, and 85.3%, 70.6%, and 61.8% in patients with two risk factors, respectively. The number of body composition risk factors could stratify the OS (Figure S8, *p* = 0.047). However, in the multivariate Cox regression model, the number of body composition risk factors was not independently associated with OS (Table S3).

Given that body composition characteristics reflect an individual’s nutritional and metabolic status, this suggests these characteristics may be more relevant to short-term prognosis. Therefore, we further conducted subgroup analyses by categorizing survival outcomes into early mortality (≤1 year) and late mortality (1 year). The Kaplan–Meier analysis revealed that patients with different numbers of body composition risk factors showed significant differences in survival for early mortality (*p* = 0.009, Figure 5A), whereas no statistically significant difference was observed for late mortality (*p* = 0.627, Figure 5B).

In the subsequent multivariate analysis, the number of body composition risk factors remained independently associated with early mortality (1 risk factor, HR: 2.02, 95% CI: 1.05–3.87, *p* = 0.035; 2 risk factors, HR: 3.32, 95% CI: 1.36–8.06, *p* = 0.008). Other variables independently associated with early mortality included non-TOs (HR: 1.61, 95% CI: 1.01–2.56, *p* = 0.046), tumor size (5 cm, HR: 2.57, 95% CI: 1.53–4.31, *p* < 0.001), α-fetoprotein (400 ng/mL, HR: 2.45, 95% CI: 1.55–3.89, *p* < 0.001), APRI (1.5, HR: 1.91, 95% CI: 1.12–3.27, *p* = 0.017), and smoking status (yes, HR: 1.72, 95% CI: 1.09–2.73, *p* = 0.021, Table 4). Variables independently associated with late death are shown in Supplementary Table S4.

### 3.5. Bootstrap Internal Validation of the PMI and SFI Cutoff Values

The sex-specific cutoffs recalculated in the complete cohort were consistent with the originally reported values. For the PMI, the bootstrap median cutoff was 4.18 cm^2^/m^2^ (2.5th–97.5th percentiles, 3.70–6.61) in women and 7.72 cm^2^/m^2^ (5.21–7.95) in men (Figure S9A,B), compared with the original cutoffs of 3.84 and 7.81 cm^2^/m^2^, respectively. The optimal-corrected AUCs of the dichotomous PMI cutoff rule were 0.524 in women and 0.557 in men (Figure S10A,B). For the combined sex-specific PMI classification, the apparent AUC decreased from 0.574 to an optimal-corrected AUC of 0.558.

For the SFI, the bootstrap median cutoff was 80.09 cm^2^/m^2^ (2.5th–97.5th percentiles, 59.68–101.13) in women and 49.16 cm^2^/m^2^ (19.58–69.79) in men, compared with the original cutoffs of 80.09 and 56.75 cm^2^/m^2^, respectively (Figure S9C,D). The corresponding optimal-corrected AUCs were 0.573 in women and 0.506 in men (Figure S10C,D). For the combined sex-specific SFI classification, the apparent AUC decreased from 0.535 to an optimal-corrected AUC of 0.513.

Using the original sex-specific cutoffs, a low PMI was associated with non-TOs in the unadjusted analysis (OR: 1.97; 95% CI: 1.40–2.77, *p* < 0.001), whereas a high SFI was associated with non-TOs, with an OR of 1.63 (95% CI: 1.10–2.42, *p* = 0.015). The corresponding optimal-corrected unadjusted ORs were 1.68 for a low PMI and 1.29 for a high SFI. Overall, the PMI showed relatively greater cutoff reproducibility than the SFI, whereas the SFI cutoff demonstrated substantial variability, particularly among men.

## 4. Discussion

Adequate assessment of surgical quality is crucial for enabling patients to benefit from surgical procedures. Textbook outcomes (TOs), as composite indicators, have been widely used and perform well in the evaluation of surgical quality, including liver surgery. Although some previous studies have explored the factors influencing the achievement of TOs after hepatectomy, these studies have focused primarily on intraoperative conditions and tumor burden, while somewhat neglecting the impact of patients’ individualized body composition characteristics on TOs. Thus, our study investigated the association between body composition and TOs in HCC patients after hepatectomy and identified body composition parameters (a low PMI and a high SFI) that were independently associated with failure to achieve TOs. An increasing number of these two factors showed graded associations with non-TOs and early mortality, but not with late mortality. These findings suggest that selected preoperative CT-derived body composition characteristics are associated predominantly with short-term outcomes after hepatectomy for HCC.

Currently, CT cross-sectional images at the L3 level are widely adopted for the assessment of body composition parameters. In our study, we measured the cross-sectional area and the mean density of various muscles and the area of adipose tissue using L3-level CT images. The areas of different muscle tissues were normalized by dividing by the square of its height. Since no prior studies have investigated the association between body composition parameters and postoperative TOs in HCC patients after hepatectomy, we determined optimal cutoff values for these parameters using ROC curve analysis based on our data. After adjusting for body composition parameters and other confounding factors by multivariate logistic regression, we finally identified a low PMI and a high SFI as independent risk factors for TO achievement in HCC patients after hepatectomy. Skeletal muscle mass reflects the nutritional status of patients to some extent. For patients with carcinoma, a low PMI may reflect reduced psoas muscle quantity in the context of disease-related catabolism and malnutrition [26]. Some previous studies have indicated that reduced skeletal muscle mass is closely associated with perioperative surgical outcomes [27,28,29,30]. Asai Y et al. reported that the low PMI was an independent controversial [30] intraoperative blood loss and have suggested rative infectious complications in patients with perihepatectomy [31] carcinoma undergoing [29] hepatectomy [13]. Batista et al. [28] explored the factors associated with postoperative complications in major intra-abdominal colorectal surgery based on PMI-defined sarcopenia and demonstrated that sarcopenia was significantly correlated with major complications and the severity of complications. Peng et al. [29] also reported that patients who underwent liver resection complications liver metastasis with low psoas muscle area had a prolonged LOS and had a higher risk of experiencing postoperative complications [31]. In the analysis for each type of TO, our conclusions were consistent with those of previous studies (Table 3). TOs, as comprehensive indicators of surgical quality assessment, cover parameters including length of hospitalization, major complications, and perioperative blood transfusion. This could, to some extent, explain why HCC patients with a low PMI were less likely to achieve TOs after hepatectomy. Although the optimal choice of abdominal muscles for assessing muscle mass remains controversial [31,32], some recent studies have suggested that the PMI may be a more robust and independent indicator for muscle mass in HCC patients [27,33,34]. This was, to some extent, consistent with our research findings. However, it is noteworthy that a low PMI alone cannot establish a diagnosis of sarcopenia in the absence of assessments of muscle strength and physical performance. Future prospective studies incorporating comprehensive assessments of muscle quantity, strength, physical performance, nutritional status, and frailty are needed to further clarify the clinical relevance of a low CT-derived PMI in patients undergoing hepatectomy for HCC. In addition, subcutaneous adiposity represents a body composition domain distinct from muscle quantity. Previous studies have reported associations between increased subcutaneous adipose tissue and postoperative wound-related complications [35], which may contribute to prolonged postoperative recovery. In our study, a prolonged LOS accounted for a greater proportion of non-TO events. Thus, it was reasonable that a high SFI was an independent risk factor for TO achievement. A low PMI may reflect reduced muscle and physiological reserve, which is consistent with the concept that reduced muscle quantity is associated with increased vulnerability to surgical stress, postoperative morbidity, and delayed recovery. The SFI reflects subcutaneous adiposity, a distinct body composition domain not captured by muscle measures alone. Increased subcutaneous adiposity may be associated with altered metabolic status, systemic inflammation, impaired wound healing, and prolonged recovery. Therefore, the associations of a low PMI and a high SFI with failure to achieve TOs are broadly consistent with existing concepts of muscle depletion- and adiposity-related surgical risk.

In the further analysis of our study, we observed a cumulative association between the number of CT-derived body composition risk factors and postoperative outcomes. The likelihood of achieving TOs decreased progressively as the number of low PMI and high SFI markers increased. These findings suggest that CT-derived body composition characteristics may reflect reduced biological and nutritional reserve and may complement conventional preoperative assessment by identifying patients who warrant more comprehensive perioperative evaluation and support. However, the present retrospective observational study cannot establish whether these characteristics are causal mediators of adverse outcomes or whether modifying the PMI or SFI would improve postoperative outcomes. Although exercise-based and nutritional interventions have been reported to modify muscle mass and adipose tissue in some cancer populations [26,36], their effectiveness, feasibility, and impact on postoperative outcomes in patients undergoing hepatectomy for HCC remain uncertain. Decisions regarding supportive interventions and the timing of surgery should be individualized, taking into account tumor burden, liver function, surgical urgency, and the potential risk of disease progression. Prospective interventional studies are needed to determine whether modification of body composition translates into improved postoperative outcomes in this population.

Interestingly, our study revealed that the cumulative burden of body composition risk factors was significantly associated with early death but not late death in HCC patients after hepatectomy. The determinants of long-term postoperative prognosis in HCC patients are inherently complex, likely involving multifaceted interactions between various factors and the intrinsic biological characteristics of the disease. Due to data limitations in our study, we only assessed body composition parameters at a single preoperative timepoint without serial measurements, which inherently limits the predictive value for long-term outcomes. Nevertheless, time-specific body composition parameters reflect the nutritional status of the organism at that time, and many studies have demonstrated the association between nutritional status and short-term prognosis [37,38,39], which may, to some extent, explain why the number of body composition risk factors affects early death in HCC patients after hepatectomy. Mortality within one year after hepatectomy is inherently heterogeneous and may result from postoperative complications, liver failure, tumor recurrence, aggressive tumor biology, or unrelated medical conditions. Because cause-specific mortality data were not consistently available in this retrospective cohort, we could not determine whether the associations with body composition markers differed by cause of death. Therefore, these CT-derived measures should be interpreted as markers of biological reserve associated with all-cause early mortality, rather than direct determinants of specific causes of death.

The PMI and SFI were emphasized because they were the two body composition measures that retained statistically significant associations with non-TOs after multivariable adjustment in the present cohort. However, neither measure was prespecified as the primary exposure. They emerged from an exploratory evaluation of multiple, partly correlated CT-derived parameters using outcome-derived cutoffs and data-dependent variable screening. Their retention in the final model may therefore reflect, at least in part, multiple testing, correlations among the candidate measures, and model-selection variability. The present findings should not be interpreted as demonstrating that the PMI or SFI is biologically, prognostically, or clinically superior to the other body composition measures examined (e.g., wall SMI, total SMI, muscle radiodensity, VFI). Rather, the PMI and SFI should be regarded as candidate markers identified in this cohort that require confirmation through prespecified analyses in independent populations.

Bootstrap internal validation provided further insight into the reproducibility of the sex-specific PMI and SFI cutoffs. The bootstrap estimates for the male PMI and female SFI were generally centered near the original values, whereas greater variability was observed for the female PMI and, more prominently, the male SFI. These findings indicate that the precise numerical thresholds may be sensitive to the study population. However, such variability does not negate the exploratory associations of a low PMI and a high SFI with the failure to achieve TOs. Rather, it supports their interpretation as candidate body composition markers while highlighting the need to externally validate and potentially recalibrate the proposed cutoffs before they are applied in clinical practice.

There were several limitations to our study. First, our study was a single-center retrospective study and might have some degree of selection bias. In the future, we plan to conduct a prospective multicenter study to validate our findings. Second, neither the PMI nor the SFI was prespecified as the primary body composition exposure. These measures were selected after exploratory evaluation of 11 candidate CT-derived parameters. Sex-specific cutoffs were derived using non-TOs as the outcome, and the same cohort was subsequently used for univariable screening, multivariable effect estimation, and construction of the PMI/SFI marker count. No formal correction for multiple comparisons was applied. Consequently, the possibility of false-positive findings, optimistic effect estimates, and instability in the selected parameters cannot be excluded. In addition, dichotomization may oversimplify potentially continuous or nonlinear associations between body composition and postoperative outcomes. Although bootstrap internal validation was performed, this analysis cannot replace validation in an independent cohort. The bootstrap results demonstrated variability in the sex-specific cutoff values and limited optimal-corrected discrimination, particularly for the SFI among men. Moreover, restricted cubic spline analyses were not performed, and dichotomization may have obscured potentially continuous or nonlinear associations. Therefore, the PMI and SFI cutoffs and their post hoc combination should remain exploratory until externally validated in larger, independent populations using prespecified analysis plans. Third, we mainly included patients with HBV-related HCC in our study, and the applicability of our conclusions to patients with HCC of other etiologies requires further validation. Fourth, muscle-related body composition parameters were derived from a single preoperative CT image at the L3 level. Although these parameters objectively characterize muscle quantity and radiodensity, they do not directly assess muscle strength or physical function. As functional measures such as handgrip strength and gait speed were unavailable, our findings reflect the associations of CT-derived muscle characteristics, rather than muscle function, with textbook outcomes and early mortality. Future prospective studies incorporating standardized functional assessments are warranted. In addition, although all segmentations were reviewed by two trained investigators, the review was performed as a consensus-based quality-control process rather than as independent duplicate segmentation. Manual corrections were not recorded at the individual-scan level. Consequently, the frequency of manual correction and study-specific interobserver agreement could not be quantified. Finally, we did not perform prespecified subgroup analyses or formal interaction testing according to hepatic functional reserve, tumor burden, or operative extent. Because some clinically relevant subgroups had limited sample sizes and outcome events, such analyses could have produced unstable estimates. Therefore, the associations observed in the overall cohort should not be assumed to be uniform across all patient subgroups. Larger multicenter studies with prespecified interaction analyses are needed to evaluate potential effect modification by liver function, tumor burden, etc.

## 5. Conclusions

In conclusion, this study clarified the body composition risk factors affecting the achievement of TOs in HCC patients after hepatectomy. An increasing number of patients with a low PMI and a high SFI showed graded associations with non-TOs and early mortality, but not with late mortality. These findings warrant validation in independent cohorts.

## Figures and Tables

**Figure 1:**
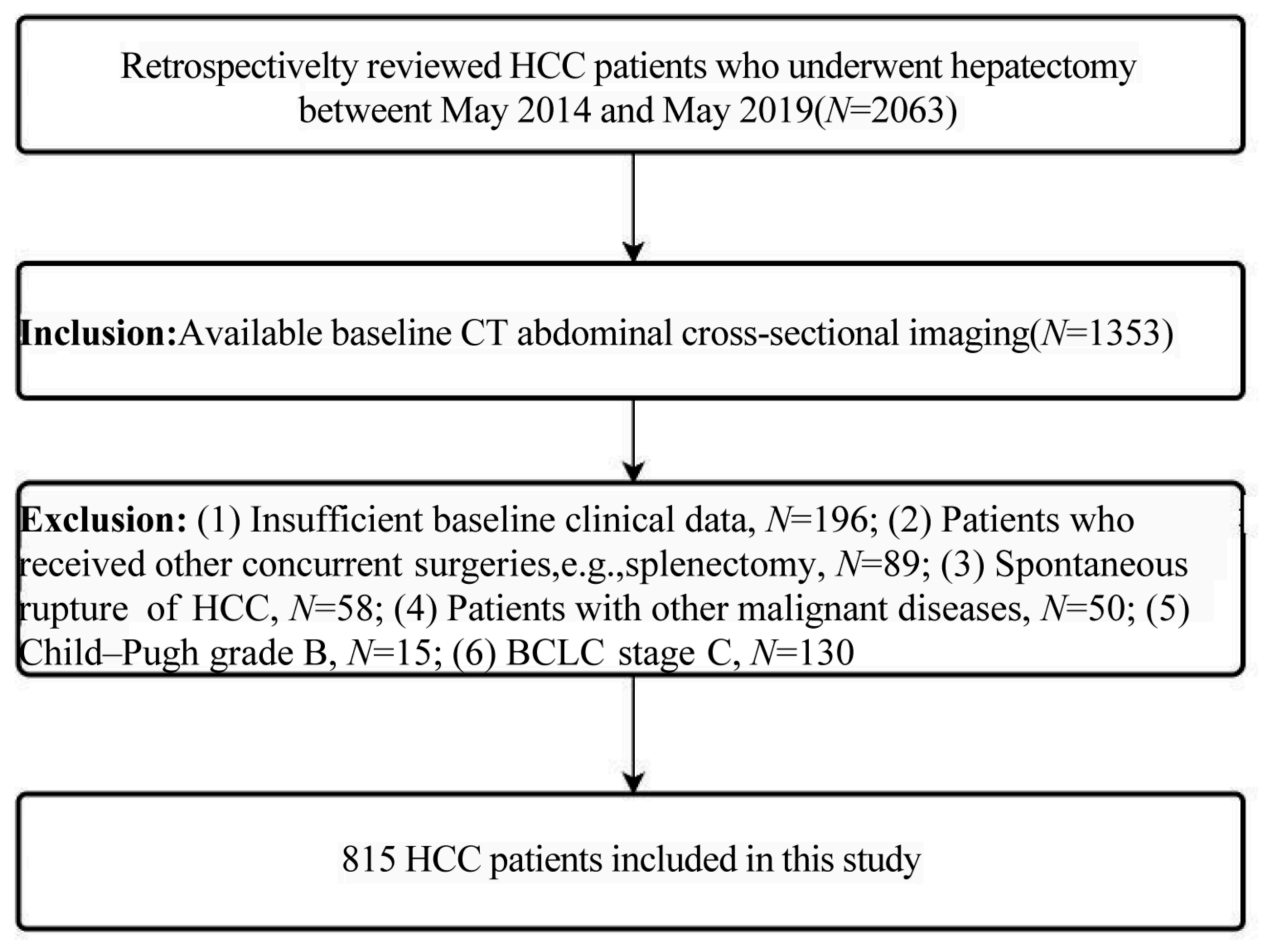
Flowchart of patient selection.

**Figure 2:**
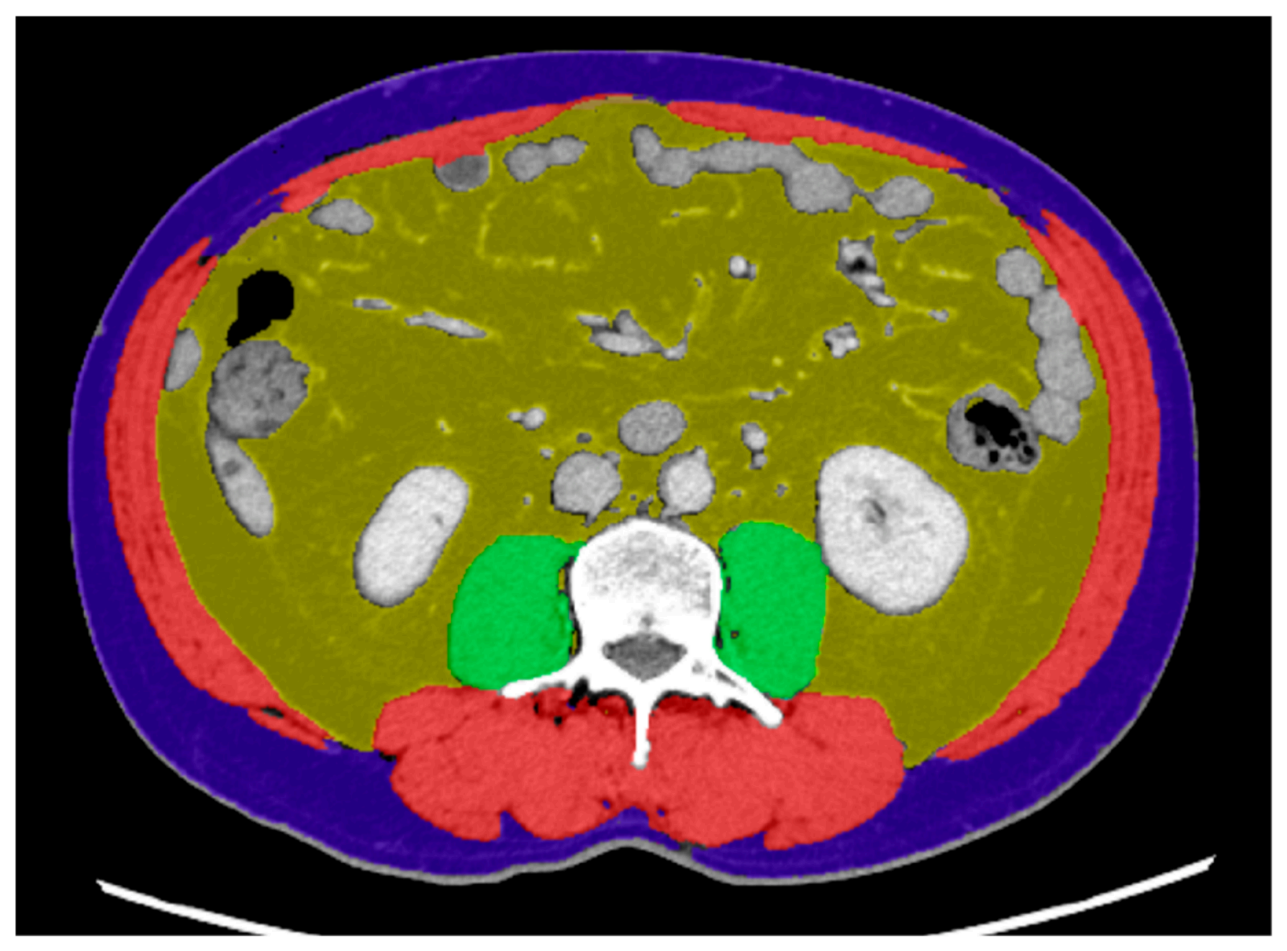
Measurement of body composition parameters based on the cross-sectional computed tomography (CT) images of the third lumbar vertebra. Wall muscle (red area); psoas muscle (green area); total muscle (red area plus green area); visceral adipose tissue (yellow area); and subcutaneous adipose tissue (blue area).

**Figure 3:**
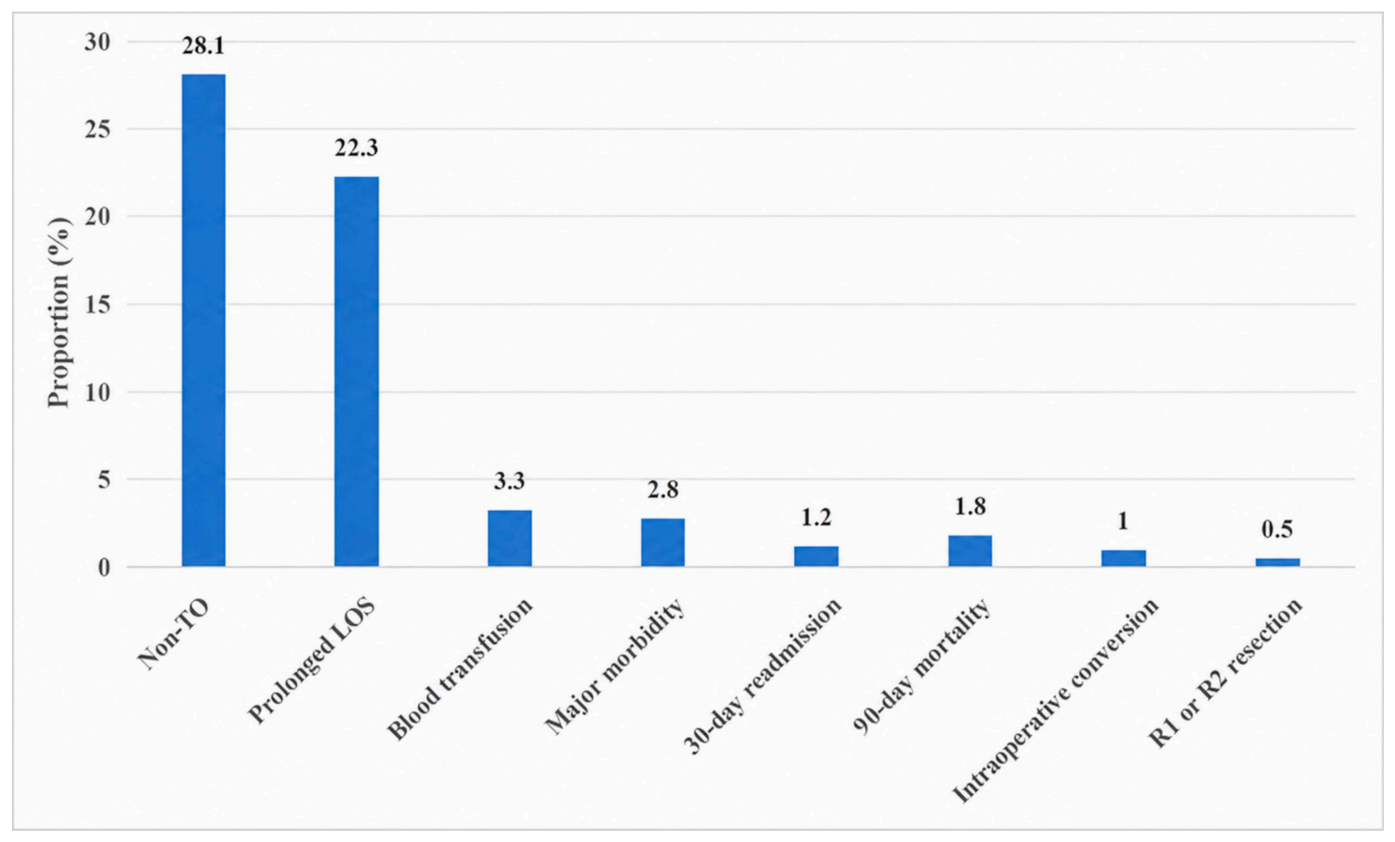
Distribution of each type of non-textbook outcome.

**Figure 4:**
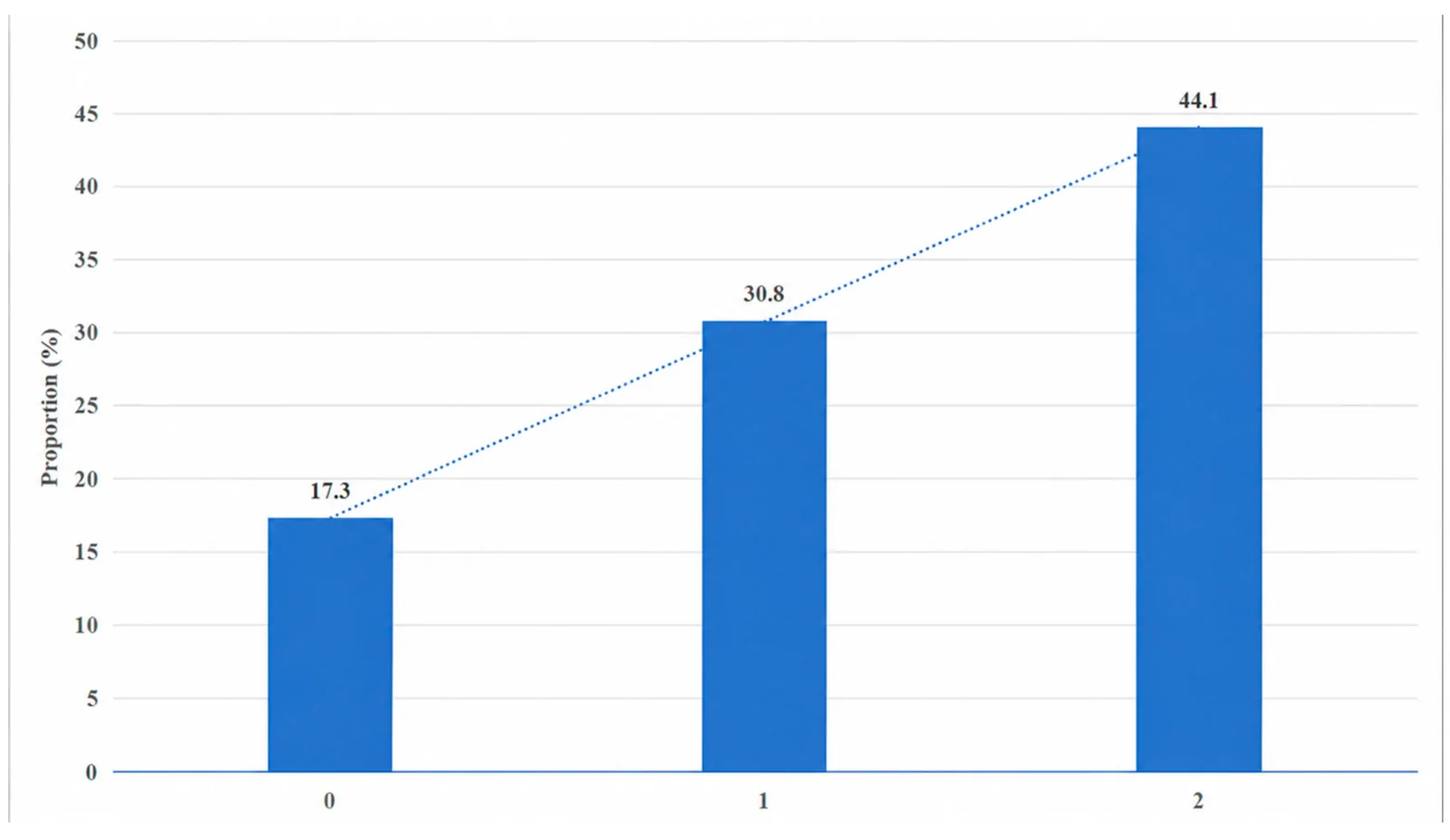
The incidence of non-textbook outcomes in patients with different numbers of body composition risk factors.

**Figure 5:**
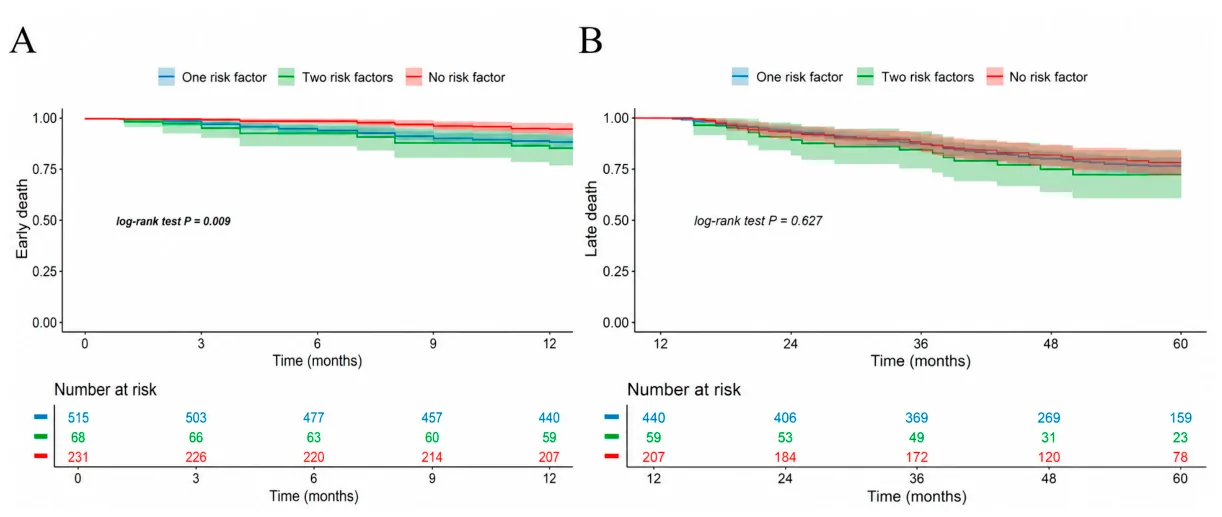
Kaplan–Meier curve for early death ((**A**) *p* = 0.009) and late death ((**B**) *p* = 0.627) by the number of body composition risk factors.

**Table 1:** Characteristics of HCC patients classified by TOs.

Characteristics	TO (*N* = 586)	Non-TO (*N* = 229)	*p*-Value
Age, years, median (IQR)	52 (46, 62)	53 (47, 62)	0.261
Gender, *N* (%)			
Male/Female	494/92 (84.3/15.7)	199/30 (86.9/13.1)	0.350
Etiology, *N* (%)			
HBV/Others	562/24 (95.9/4.1)	223/6 (97.4/2.6)	0.315
HBV load, >2000 IU/L, *N* (%)	201 (34.3)	88 (38.4)	0.268
Smoking status, *N* (%)	295 (50.3)	109 (47.6)	0.481
Alcohol consumption, *N* (%)	241 (41.1)	84 (36.7)	0.244
Hypertension, *N* (%)	102 (17.4)	38 (16.6)	0.782
Diabetes, *N* (%)	41 (7.0)	25 (10.9)	0.065
Body mass index, kg/m^2^, median (IQR)	23.3 (21.2, 25.3)	23.1 (21.3, 25.2)	0.657
BCLC stage, *N* (%)			
0/A/B	50/505/31 (8.5/86.2/5.3)	8/201/20 (3.5/87.8/8.7)	0.011
Tumor size, cm, median (IQR)	4.2 (2.8, 6.6)	5.5 (3.7, 8.6)	<0.001
Single tumor, *N* (%)	541 (92.3)	207 (90.4)	0.368
α-fetoprotein, >400 ng/mL, *N* (%)	199 (34.0)	98 (42.8)	0.018
Total bilirubin (μmol/L), median (IQR)	13.5 (10.6, 17.8)	13.6 (10.9, 17.7)	0.788
ALT (U/L), median (IQR)	33 (23, 48)	37 (26, 54)	0.003
AST (U/L), median (IQR)	32 (26, 43)	39 (29, 57)	<0.001
Albumin (g/L), median (IQR)	43.6 (41.1, 46.1)	42.1 (39.1, 44.4)	<0.001
Platelet count, >100 × 10^9^/L, *N* (%)	429 (73.2)	171 (74.7)	0.670
APRI, median (IQR)	0.6 (0.4, 1.0)	0.8 (0.5, 1.3)	0.004
FIB-4, median (IQR)	2.4 (1.6, 3.7)	2.6 (1.7, 4.3)	0.045
PNI, median (IQR)	51.3 (47.7, 54.4)	49.0 (45.5, 52.6)	<0.001
ALBI grade I/II, *N* (%)	518/68 (88.4/11.6)	176/53 (76.9/23.1)	<0.001
Laparoscopic/open hepatectomy, *N* (%)	147/439 (25.1/74.9)	53/176 (23.1/76.9)	0.563
Major hepatectomy, *N* (%)	190/396 (32.4/67.6)	115/114 (50.2, 49.8)	<0.001
ASA grade, ≥3, *N* (%)	63 (10.8)	29 (12.7)	0.438
Intraoperative blood loss, median (IQR)	200 (100, 300)	300 (150, 500)	<0.001
Length of hospital stay, median (IQR)	8 (7, 10)	14 (12, 16)	<0.001
**Body composition**			
Imaging-to-surgery interval in days, median (IQR)	4 (2, 9)	5 (2, 8)	0.119
PMI, cm^2^/m^2^, high/low, *N* (%)	235/351 (40.1/59.9)	58/171 (25.3/74.7)	<0.001
Psoas SMD, HU, high/low, *N* (%)	165/421 (28.2/71.8)	49/180 (21.4/78.6)	0.049
Wall SMI, cm^2^/m^2^, high/low, *N* (%)	234/352 (39.9/60.1)	86/143 (37.6/62.4)	0.532
Wall SMD, HU, high/low, *N* (%)	118/468 (20.1/79.9)	29/200 (12.7/87.3)	0.013
Total SMI, cm^2^/m^2^, high/low, *N* (%)	280/306 (47.8/52.2)	101/128 (44.1/55.9)	0.344
Total SMD, HU, high/low, *N* (%)	243/343 (41.5/58.5)	93/136 (40.6/59.4)	0.823
SFI, cm^2^/m^2^, high/low, *N* (%)	82/504 (14.0/86.0)	48/181 (21.0/79.0)	0.015
VSI, cm^2^/m^2^, high/low, *N* (%)	194/392 (33.1/66.9)	90/139 (39.3/60.7)	0.095
Visceral-fat-to-muscle ratio, high/low, *N* (%)	159/427 (27.1/72.9)	78/151 (34.1/65.9)	0.050
Subcutaneous-fat-to-muscle ratio, high/low, *N* (%)	111/475 (18.9/81.1)	61/168 (26.6/73.4)	0.016
Visceral-to-subcutaneous-fat ratio, high/low, *N* (%)	436/150 (74.4/25.6)	175/54 (76.4/23.6)	0.550

Abbreviations: TO, textbook outcome; HBV, hepatitis B virus; BCLC stage, Barcelona Clinic Liver Cancer stage; ALT, alanine aminotransferase; AST, aspartate aminotransferase; APRI, aspartate aminotransferase-to-platelet ratio index; FIB-4, fibrosis-4 index; PNI, prognostic nutritional index; ALBI grade, albumin-bilirubin grade.

**Table 2:** Factors associated with non-TOs in HCC patients receiving hepatectomy.

		Univariable			Multivariable		
Variables		OR	95% CI	*p*-Value	OR	95% CI	*p*-Value
BCLC stage							
0		Ref					
A		2.49	1.16–5.34	0.019			
B		4.03	1.58–10.26	0.003			
Tumor size, cm	>5 cm vs. ≤5 cm	1.93	1.42–2.63	<0.001			
α-fetoprotein, ng/mL	>400 ng/mL vs. ≤400 ng/mL	1.46	1.06–1.99	0.019	1.41	1.01–1.97	0.046
ALT, U/L	>40 U/L vs. ≤40 U/L	1.45	1.06–1.98	0.019			
AST, U/L	>40 U/L vs. ≤40 U/L	2.15	1.57–2.94	<0.001	1.60	1.14–2.26	0.007
Albumin, g/L	>40 g/L vs. ≤40 g/L	0.43	0.30–0.62	<0.001	0.53	0.36–0.77	<0.001
APRI	>1.5 vs. ≤1.5	2.12	1.39–3.25	<0.001			
PNI	>45 vs. ≤45	0.42	0.28–0.64	<0.001			
ALBI grade	II vs. I	2.29	1.54–3.42	<0.001			
Major hepatectomy	Yes vs. No	2.10	1.54–2.87	<0.001	1.61	1.14–2.24	0.006
Intraoperative blood loss	>400 mL vs. ≤400 mL	2.38	1.63–3.49	<0.001	1.66	1.10–2.50	0.016
PMI, cm^2^/m^2^	Low vs. High	1.97	1.40–2.78	<0.001	1.91	1.33–2.73	<0.001
Psoas SMD, HU	Low vs. High	1.44	1.01–2.07	0.049			
Wall SMD, HU	Low vs. High	1.74	1.12–2.70	0.013			
SFI, cm^2^/m^2^	High vs. Low	1.63	1.10–2.42	0.015	1.91	1.25–2.92	0.003
Subcutaneous-fat-to-muscle ratio	High vs. Low	1.55	1.09–2.22	0.016			

**Table 3:** Comparison of each type of non-TO between patients with or without body composition risk factors.

Distribution of Each Type of Non-TO	0 (*N* = 231)	1/2 (*N* = 583)	*p*-Value
Postoperative 30-day major morbidity	2 (0.9%)	21 (3.6%)	0.034
Postoperative 30-day readmission	0	10 (1.7%)	0.045
Postoperative 90-day mortality	0	15 (2.6%)	0.014
Perioperative blood transfusion	3 (1.3%)	24 (4.1%)	0.043
R1 or R2 resection	0	4 (0.7%)	0.207
Intraoperative conversion of LLR	0	8 (1.4%)	0.074
Prolonged length of hospital stay	37 (16.0%)	145 (24.8%)	0.006

**Table 4:** Variables independently associated with early death (within 1 year) in HCC patients after hepatectomy.

		Univariable			Multivariable		
Variables		HR	95% CI	*p*-Value	HR	95% CI	*p*-Value
Number of risk factors							
0		Ref					
1		2.43	1.27–4.62	0.007	2.02	1.05–3.87	0.035
2		3.16	1.34–7.45	0.008	3.32	1.36–8.06	0.008
Non-TOs (vs. TOs)		2.43	1.56–3.78	<0.001	1.61	1.01–2.56	0.046
Tumor size, cm	>5 cm vs. ≤5 cm	3.77	2.29–6.21	<0.001	2.57	1.53–4.31	<0.001
α-fetoprotein, ng/mL	>400 ng/mL vs. ≤400 ng/mL	2.56	1.64–4.01	<0.001	2.45	1.55–3.89	<0.001
AST, U/L	>40 U/L vs. ≤40U/L	2.71	1.74–4.24	<0.001			
Albumin, g/L	>40 g/L vs. ≤40 g/L	0.42	0.27–0.67	<0.001			
APRI	>1.5 vs. ≤1.5	2.37	1.41–3.97	0.001	1.91	1.12–3.27	0.017
PNI	>45 vs. ≤45	0.55	0.32–0.93	0.027			
ALBI grade	II vs. I	2.41	1.47–3.93	0.001			
Major hepatectomy	Yes vs. No	2.24	1.44–3.49	<0.001			
Intraoperative blood loss	>400 mL vs. ≤400 mL	2.35	1.45–3.79	<0.001			
Smoking status	Yes vs. No	1.59	1.01–2.50	0.045	1.72	1.09–2.73	0.021

## Data Availability

The original contributions presented in this study are included in the article. Further inquiries can be directed to the corresponding author.

## Supplementary Materials

The following supporting information can be downloaded at: https://www.mdpi.com/article/10.3390/jcm15197547/s1, Figure S1: Distribution of the imaging-to-surgery interval in the study cohort; Figure S2: Sex-specific cutoff values of the PMI (male/female, A/B) and psoas SMD (male/female, C/D); Figure S3: Sex-specific cutoff values for wall SMI (male/female, A/B) and wall SMD (male/female, C/D); Figure S4: Sex-specific cutoff values of total SMI (male/female, A/B) and total SMD (male/female, C/D); Figure S5: Sex-specific cutoff values of the SFI (male/female, A/B) and VSI (male/female, C/D); Figure S6: Sex-specific cutoff values for the subcutaneous-fat-to-muscle ratio (male/female, A/B) and the visceral-fat-to-muscle ratio (male/female, C/D); Figure S7: Sex-specific cutoff values for the visceral-to-subcutaneous-fat ratio (male/female, A/B); Figure S8: Kaplan–Meier curve for overall survival by the number of body composition risk factors (*p* = 0.047); Figure S9: Bootstrap distributions of the sex-specific PMI and SFI cutoff values; Figure S10: Bootstrap internal validation of sex-specific PMI and SFI cutoff rules for predicting failure to achieve textbook outcomes; Table S1: Baseline body composition variables; Table S2: The number of body composition risk factors and Non-TO; Table S3: Factors associated with OS in HCC patients receiving hepatectomy; Table S4: Factors associated with late death in HCC patients receiving hepatectomy.
