# Supplementary material for: Associations of CT-Derived Body Composition with Textbook Outcomes and Early Death After Hepatectomy for Hepatocellular Carcinoma
Source: J Clin Med. 2026 Sep 28;15(19):7547. doi: 10.3390/jcm15197547 (PMC13666347; doi:10.3390/jcm15197547)
Supplement: Supplementary file 1 [file jcm-15-07547-s001.zip › jcm-4461425-supplementary.pdf]

Table S1: Baseline body composition variables

Body composition variables	Male (*N*=693)	Female (*N*=122)	*p*-value
PMI, cm2/m2, median (IQR)	6.88(5.80,8.03)	4.63(3.87,5.48)	<0.001
Psoas SMD, HU, median (IQR)	52.27(47.01,57.22)	53.12(47.16,58.63)	0.578
Wall SMI, cm2/m2, median (IQR)	43.82(40.01,47.86)	35.78(31.53,39.44)	<0.001
Wall SMD, HU, median (IQR)	43.63(36.77,48.67)	38.53(32.53,46.02)	<0.001
Total SMI, cm2/m2, median (IQR)	50.97(46.55,55.62)	40.24(36.17,44.48)	<0.001
Total SMD, HU, median (IQR)	44.85(38.77,49.81)	40.51(34.63,47.15)	<0.001
SFI, cm2/m2, median (IQR)	36.10(22.70,48.11)	65.76(43.75,81.24)	<0.001
VSI, cm2/m2, median (IQR)	35.18(18.69,56.36)	32.12(20.61,48.57)	0.316
Visceral fat to muscle ratio, median (IQR)	0.68(0.37,1.10)	0.83(0.48,1.22)	<0.001
Subcutaneous fat to muscle ratio, median (IQR)	0.71(0.46,0.93)	1.52(1.12,1.91)	0.018
Visceral to subcutaneous fat ratio, median (IQR)	0.97(0.72,1.26)	0.49(0.37,0.68)	<0.001

Abbreviations: IQR, interquartile range; HU, Hounsfield unit; PMI, psoas muscle index; SMD, skeletal muscle density; SMI, skeletal muscle index; SFI, subcutaneous fat index; VSI, visceral fat index.

Table S2: The number of body composition risk factors and Non-TO

			**Univariable**			**Multivariable**	
**Variables**		**OR**	**95%CI**	***p* value**	**OR**	**95%CI**	***p* value**
Number of risk factors							
0		Ref					
1		2.13	1.44-3.14	<0.001	1.99	1.33-2.97	<0.001
2		3.77	2.10-6.78	<0.001	3.54	1.91-6.58	<0.001
BCLC stage							
0		Ref					
A		2.49	1.16-5.34	0.019			
B		4.03	1.58-10.26	0.003			
Tumor size, cm	>5 cm *vs.* ≤5 cm	1.93	1.42-2.63	<0.001			
α-fetoprotein, ng/ml	>400 ng/ml *vs.* ≤400 ng/ml	1.46	1.06-1.99	0.019	1.41	1.01-1.97	0.047
ALT, U/L	>40 U/L *vs.* ≤40 U/L	1.45	1.06-1.98	0.019			
AST, U/L	>40 U/L *vs.* ≤40 U/L	2.15	1.57-2.94	<0.001	1.60	1.13-2.25	0.008
Albumin, g/L	>40 g/L *vs.* ≤40 g/L	0.43	0.30-0.62	<0.001	0.53	0.36-0.77	<0.001
APRI	>1.5 *vs.*≤1.5	2.12	1.39-3.25	<0.001			
PNI	>45 *vs.* ≤45	0.42	0.28-0.64	<0.001			
ALBI grade	II *vs.* I	2.29	1.54-3.42	<0.001			
Major hepatectomy	Yes *vs.* No	2.10	1.54-2.87	<0.001	1.60	1.14-2.24	0.006
Intraoperative blood loss	>400 ml *vs.* ≤400 ml	2.38	1.63-3.49	<0.001	1.67	1.10-2.51	0.015

Table S3: Factors associated with OS in HCC patients receiving hepatectomy.

		Univariable			Multivariable		
Variables		HR	95%CI	*p* value	HR	95%CI	*p* value
Number of risk factors							
0		Ref					
1		1.37	1.00-1.89	0.050			
2		1.73	1.07-2.80	0.024			
Non-TO (vs. TO)		1.94	1.49-2.53	<0.001	1.46	1.11-1.92	0.006
BCLC stage							
0		Ref			Ref		
A		4.50	1.67-12.11	0.003	2.79	1.02-7.61	0.046
B		11.14	3.91-31.78	<0.001	6.00	2.06-17.45	<0.001
Single tumor	No *vs.* Yes	2.01	1.38-2.94	<0.001			
Tumor size, cm	>5 cm *vs.* ≤5 cm	2.67	2.04-3.50	<0.001	1.89	1.42-2.52	<0.001
Laparoscopic hepatectomy	No *vs.* Yes	1.39	1.01-1.93	0.048			
α-fetoprotein, ng/ml	>400 ng/ml vs. ≤400 ng/ml	1.89	1.46-2.46	<0.001	1.57	1.20-2.06	<0.001
HBV-DNA load, IU/L	>2000 IU/L *vs.* ≤2000 IU/L	1.54	1.19-2.00	0.001			
AST, U/L	>40 U/L *vs.* ≤40 U/L	2.22	1.71-2.88	<0.001	1.43	1.08-1.90	0.012
Albumin, g/L	>40 g/L *vs.* ≤40 g/L	0.52	0.39-0.69	<0.001			
APRI	>1.5 *vs.* ≤1.5	2.01	1.45-2.77	<0.001			
FIB-4	>4.0 *vs.* ≤4.0	1.65	1.25-2.17	<0.001	1.42	1.06-1.90	0.019
PNI	>45 *vs.* ≤45	0.52	0.38-0.71	<0.001			
ALBI grade	II *vs.* I	2.03	1.49-2.76	<0.001	1.54	1.12-2.12	0.008
Major hepatectomy	Yes *vs.* No	1.85	1.43-2.40	<0.001			
Intraoperative blood loss	>400 ml *vs.* ≤400 ml	1.73	1.27-2.36	<0.001			

Table S4: Factors associated with late death in HCC patients receiving hepatectomy.

		Univariable			Multivariable		
Variables		HR	95%CI	*p* value	HR	95%CI	*p* value
Non-TO (vs. TO)		1.71	1.23-2.39	0.002	1.41	1.01-1.98	0.044
BCLC stage							
0		Ref			Ref		
A		2.94	1.08-7.94	0.034	1.97	0.71-5.45	0.193
B		9.20	3.14-26.91	<0.001	5.59	1.85-16.86	0.002
Single tumor	No *vs.* Yes	2.39	1.52-3.77	<0.001			
Tumor size, cm	>5 cm *vs.* ≤5 cm	2.27	1.64-3.15	<0.001	1.82	1.30-2.56	<0.001
α-fetoprotein, ng/ml	>400 ng/ml *vs.* ≤400 ng/ml	1.61	1.16-2.23	0.004			
HBV-DNA load, IU/L	>2000 IU/L *vs.* ≤2000 IU/L	1.69	1.22-2.33	0.002	1.39	1.01-1.93	0.049
AST, U/L	>40 U/L *vs.* ≤40 U/L	1.99	1.44-2.76	<0.001			
Albumin, g/L	>40 g/L vs. ≤40 g/L	0.58	0.41-0.83	0.003			
APRI	>1.5 vs. ≤1.5	1.82	1.20-2.76	0.005			
FIB-4	>4.0 vs. ≤4.0	1.69	1.20-2.39	0.003	1.61	1.13-2.29	0.009
PNI	>45 vs. ≤45	0.50	0.34-0.75	<0.001	0.63	0.42-0.95	0.029
ALBI grade	II vs. I	1.83	1.23-2.73	0.003			
Major hepatectomy	Yes vs. No	1.67	1.21-2.31	0.002			

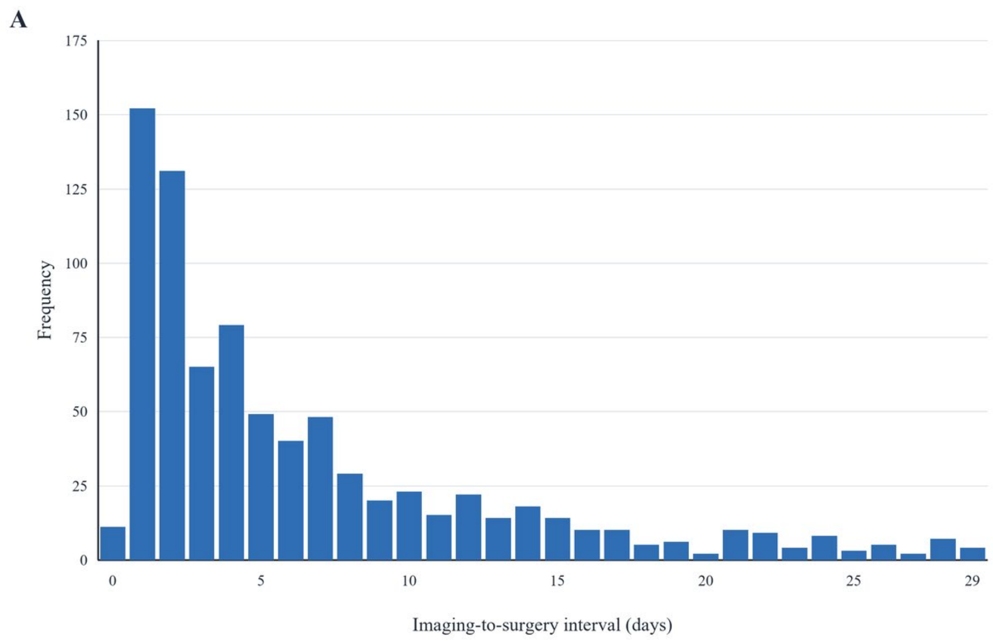

Figure S1 Distribution of the imaging-to-surgery interval in the study cohort.

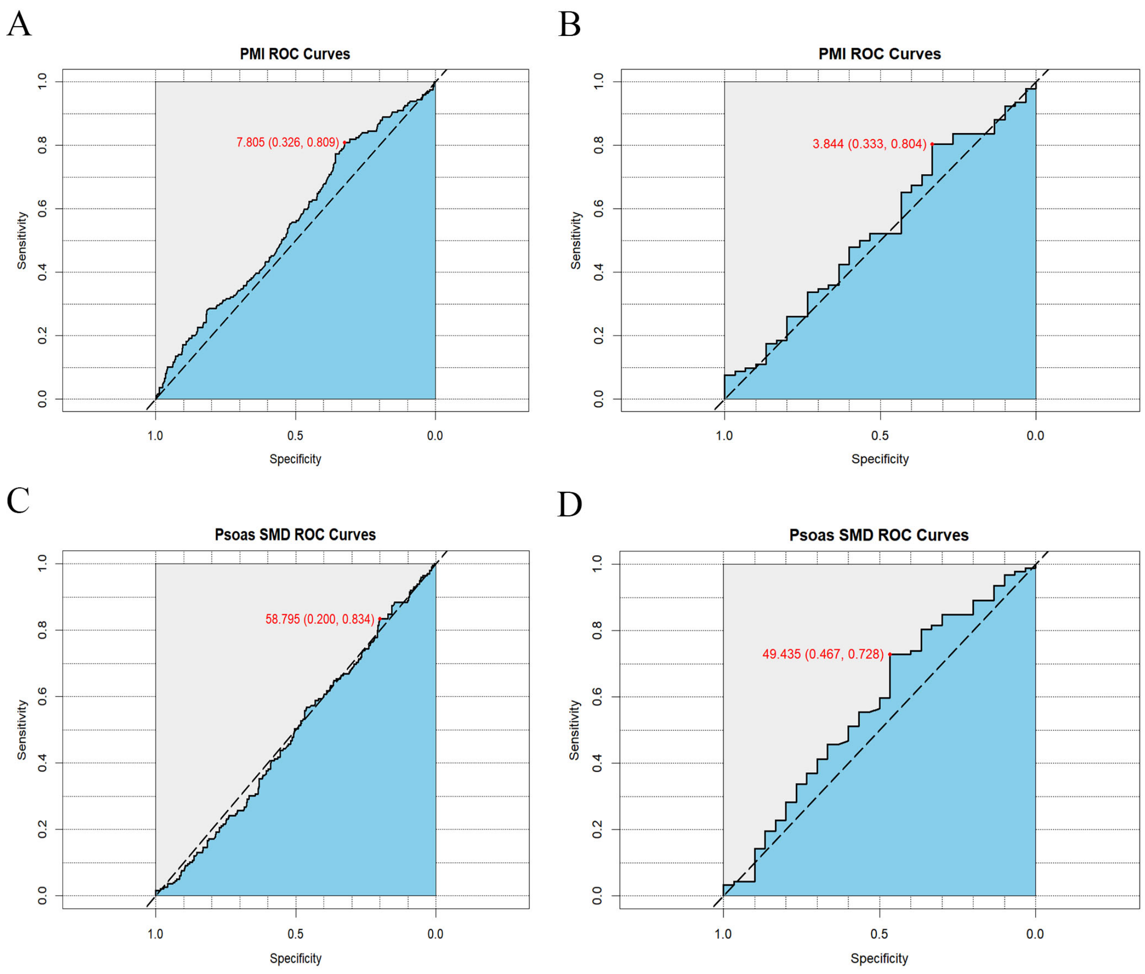

Figure S2 Sex-specific cutoff values of PMI (male/female, A/B) and psoas SMD (male/female, C/D).

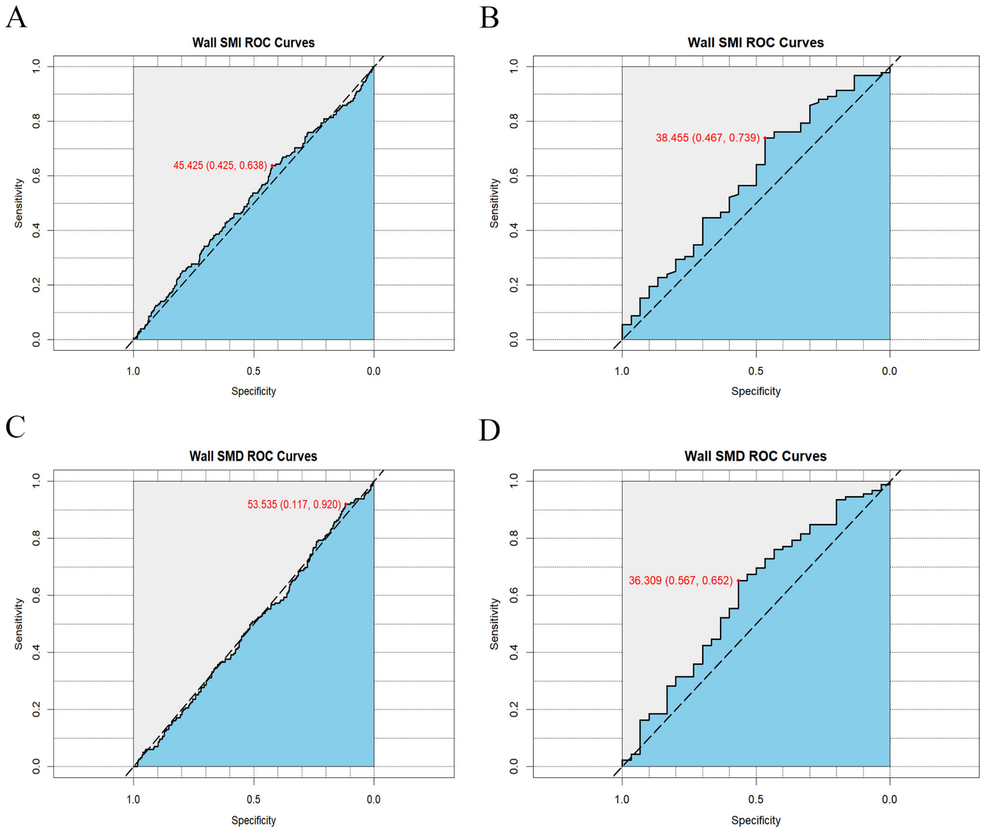

Figure S3 Sex-specific cutoff values of wall SMI (male/female, A/B) and wall SMD (male/female, C/D).

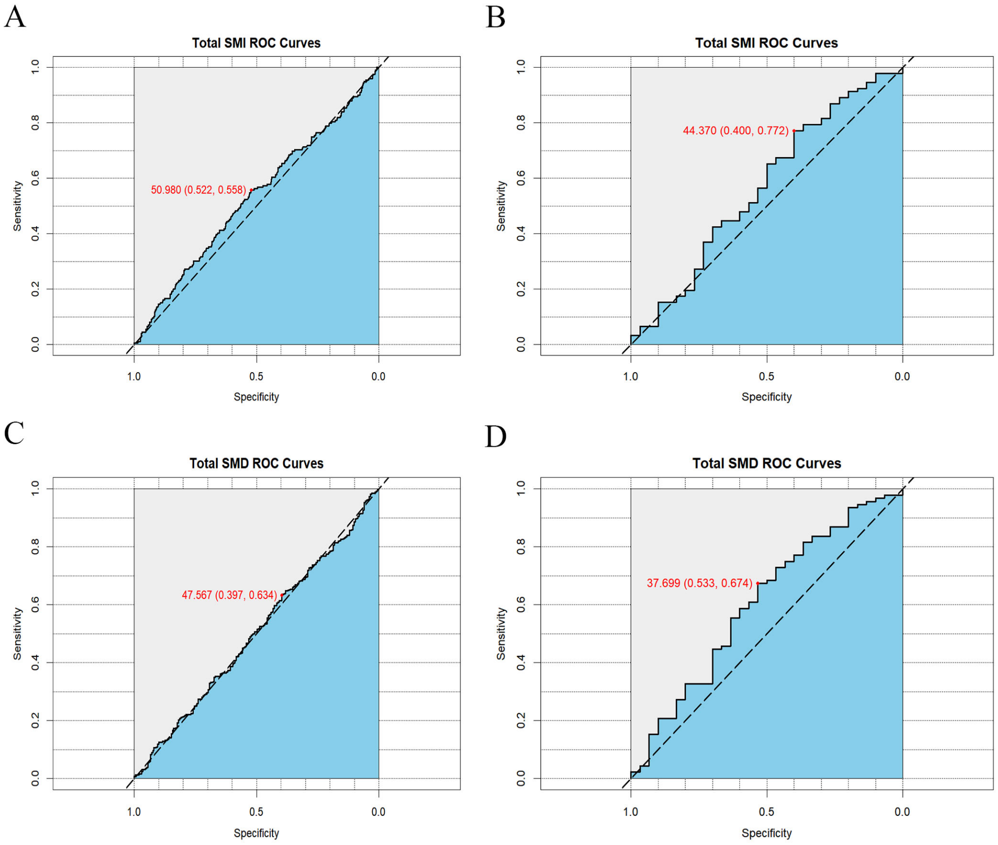

Figure S4 Sex-specific cutoff values of total SMI (male/female, A/B) and total SMD (male/female, C/D).

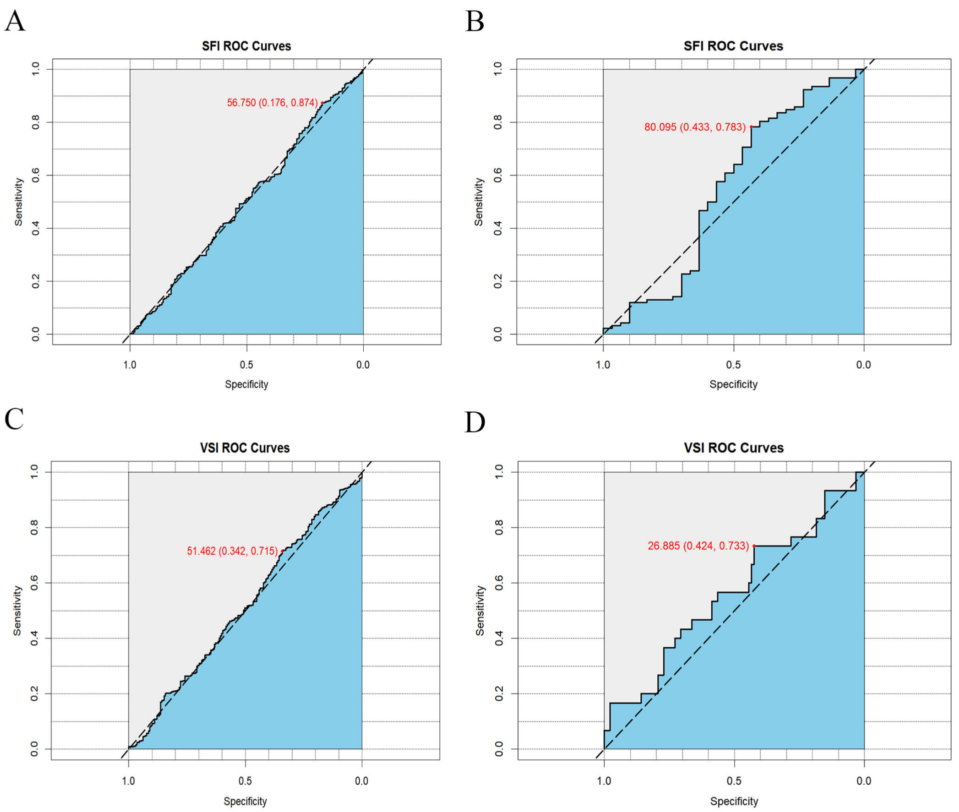

Figure S5 Sex-specific cutoff values of SFI (male/female, A/B) and VSI (male/female, C/D).

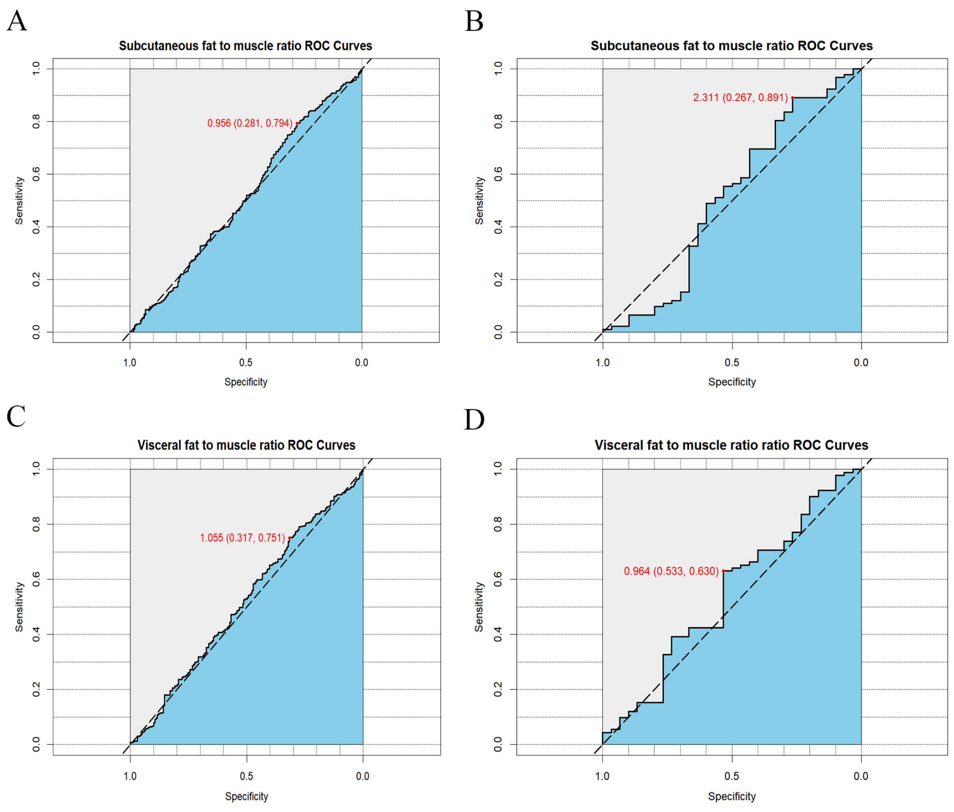

Figure S6 Sex-specific cutoff values of the subcutaneous fat to muscle ratio (male/female, A/B) and the visceral fat to muscle ratio (male/female, C/D).

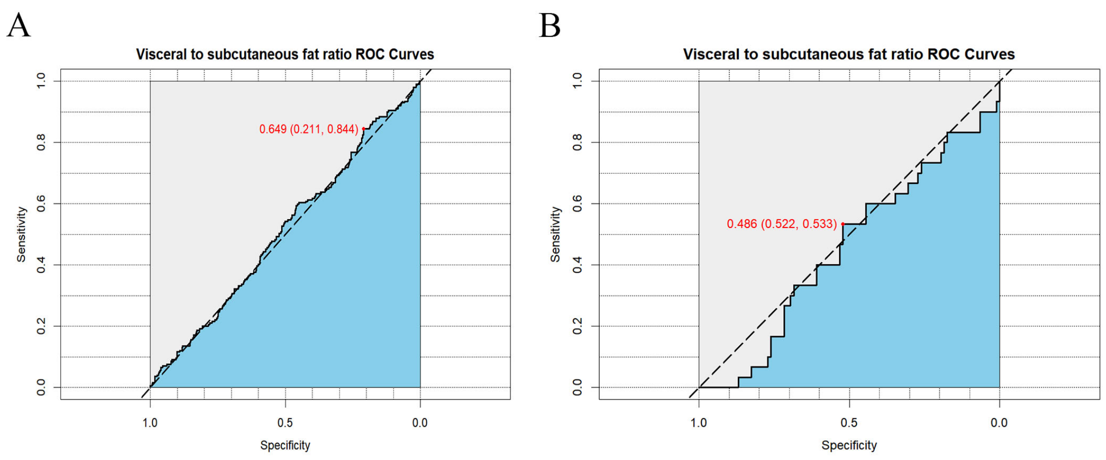

Figure S7 Sex-specific cutoff values of the visceral to subcutaneous fat ratio (male/female, A/B).

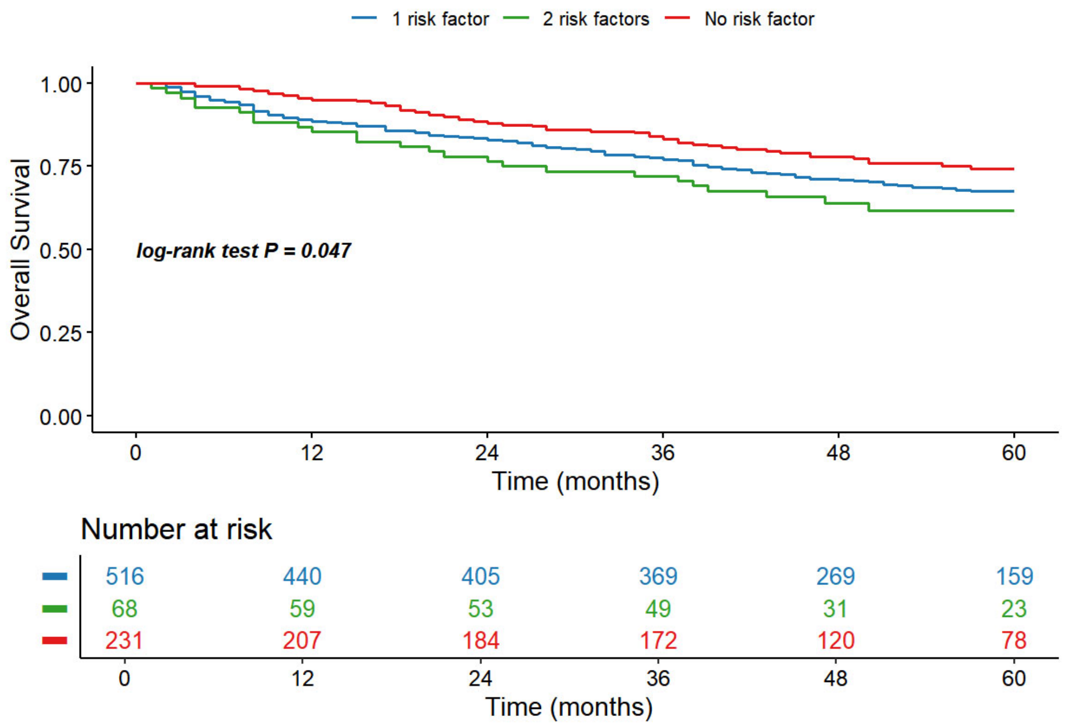

Figure S8 Kaplan–Meier curve for overall survival by the number of body composition risk factors (p=0.047).

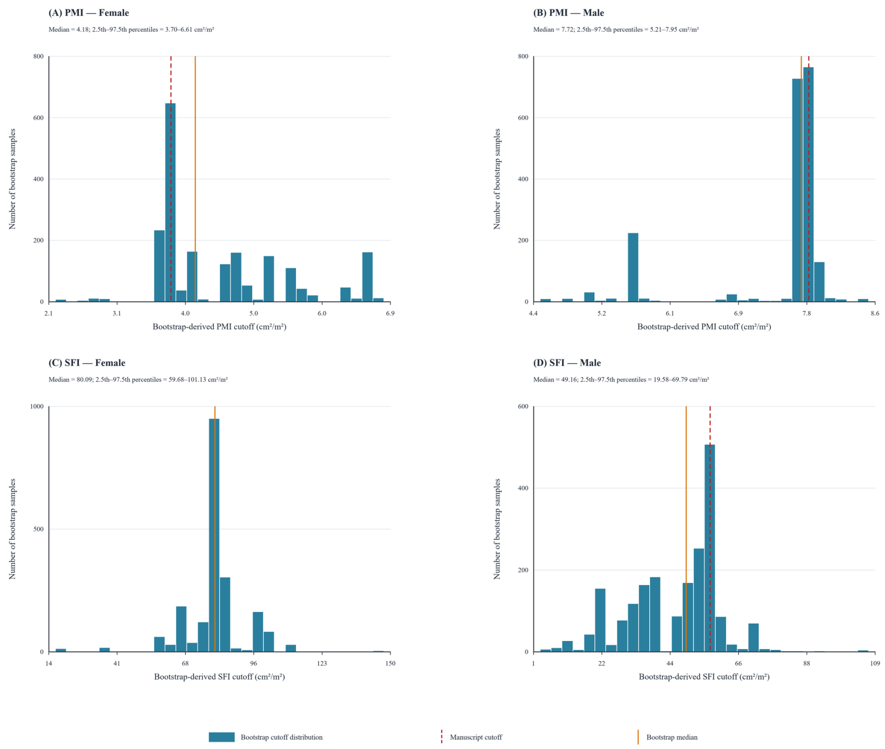

Figure S9 Bootstrap distributions of the sex-specific PMI and SFI cut-off values.

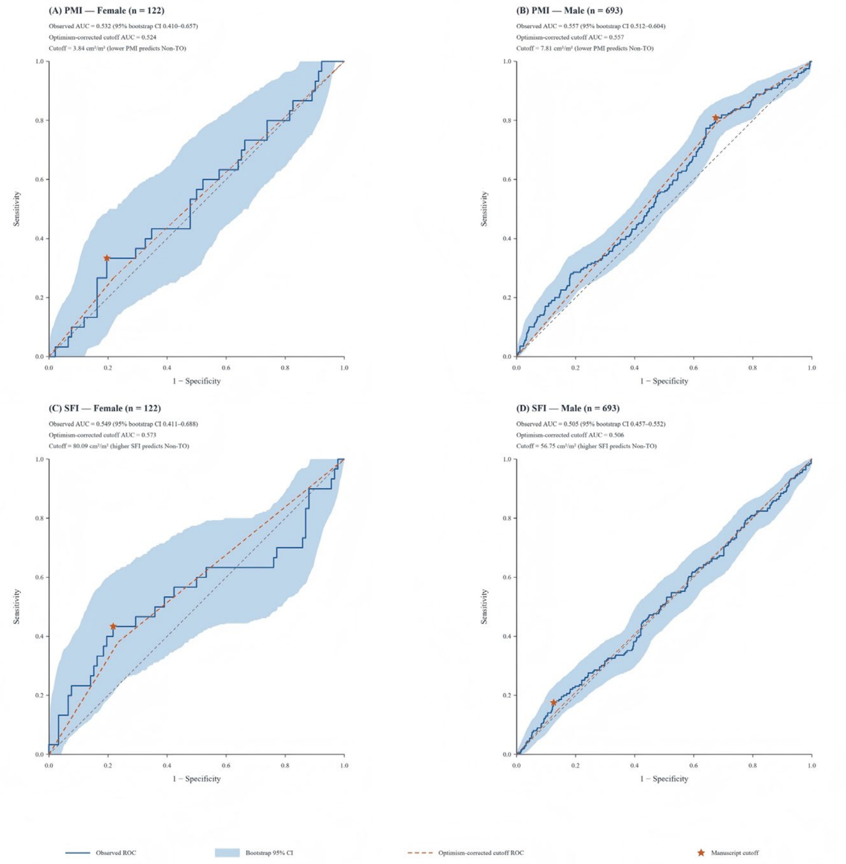

Figure S10 Bootstrap internal validation of sex-specific PMI and SFI cutoff rules for predicting failure to achieve textbook outcome.
